# Canadian CT head rule and New Orleans Criteria in mild traumatic brain injury: comparison at a tertiary referral hospital in Japan

**DOI:** 10.1186/s40064-016-1781-9

**Published:** 2016-02-25

**Authors:** Daddy Mata-Mbemba, Shunji Mugikura, Atsuhiro Nakagawa, Takaki Murata, Yumiko Kato, Yasuko Tatewaki, Kei Takase, Shigeki Kushimoto, Teiji Tominaga, Shoki Takahashi

**Affiliations:** Department of Diagnostic Radiology, Graduate School of Medicine, Tohoku University, 1-1 Seiryo-machi, Aoba-ku, Sendai, 980-8574 Japan; Department of Neurosurgery, Graduate School of Medicine, Tohoku University, Sendai, Japan; Division of Emergency and Critical Care Medicine, Graduate School of Medicine, Tohoku University, Sendai, Japan

**Keywords:** Canadian computed tomography (CT) head rule, New Orleans Criteria, Computed tomography (CT), Traumatic brain injury, Mild traumatic brain injury

## Abstract

We compared Canadian computed tomography (CT) head rule (CCHR) and New Orleans Criteria (NOC) in predicting important CT findings in patients with mild traumatic brain injury (TBI). We included 142 consecutive patients with mild TBI [Glasgow coma scale (GCS) 13–15] who showed at least one of the risk factors stated in the CCHR or the NOC. We introduced two scores: a Canadian from the CCHR and a New Orleans from the NOC. A patient’s score represented a sum of the number of positive items. We examined the relationship between scores or items and the presence of important CT findings. Only the Canadian was significantly associated with important CT findings in multivariate analyses and showed higher area under the receiver operating characteristic curve (AUC) either in all 142 patients (GCS 13–15: P = 0.0130; AUC = 0.69) or in the 67 with a GCS = 15 (P = 0.0128, AUC = 0.73). Of items, “>60 years” or “≥65 years” included in either guideline was the strongest predictor of important CT finding, followed by “GCS < 15 after 2 h” included only in the CCHR. In a tertiary referral hospital in Japan, CCHR had higher performance than the NOC in predicting important CT findings.

**Electronic supplementary material:**

The online version of this article (doi:10.1186/s40064-016-1781-9) contains supplementary material, which is available to authorized users.

## Background

Mild traumatic brain injury (TBI) is a common neurological disorder in western countries with an estimated incidence of 100–300 per 100,000 people (Cassidy et al. 2004). Mild TBI is commonly defined as a blunt injury to the head that results in a normal or minimally altered level of consciousness in the patient at presentation to the emergency department i.e., a Glasgow Coma scale (GCS) score of 13–15, and loss of consciousness for ≤15 min, or posttraumatic amnesia for ≤60 min, or both (Carroll et al. 2004). A GCS of 15 out of 15 suggests normal neurological function.

After mild TBI, intracranial complications are sometimes detected on computed tomography (CT) requiring hospitalization or neurosurgical intervention (important CT findings) (Fabbri et al. 2004; Af Geijerstam and Britton 2005). In that sense, CT plays a crucial role for reliable and rapid diagnosis of such complications (Mata-Mbemba et al. 2014, 2015). However, excessive use of CT increases unnecessary irradiation, while overly conservative usage can lead to missing life-threatening lesions.

For the purpose of proper indication without unnecessary use of CT examination, a number of clinical guidelines have been proposed in patients with mild TBI. In their review article of published clinical guidelines for predicting clinically important CT findings in patients with mild TBI, Harnan et al. (2011) reported that the Canadian CT Head Rule (CCHR) and the New Orleans Criteria (NOC) are the most frequently used guidelines (Stiell et al. 2001; Haydel et al. 2000). Recently, the CCHR and the NOC have been compared and a good balance between the sensitivity and the specificity of the CCHR over the NOC in predicting important CT findings was reported in a number of western countries (Smits et al. 2005; Stiell et al. 2005; Papa et al. 2012). However, this has not been done in Japan, which has the highest number of CT scans and presumably the highest risk for cancer from diagnostic X-rays in the world (González and Darby 2004; Nakajima et al. 2008).

In this study, we aimed to compare the performance of the CCHR and NOC guidelines in predicting important CT findings in Japanese patients with mild TBI, by introducing two scoring systems derived from the CCHR or the NOC in an attempt to weigh the contribution of individual clinical items to the overall performance of each guideline, which has never been investigated to our knowledge.

## Methods

### Patients

In order to confirm that CCHR had higher performance than the NOC not only in western countries but also in Japan, we followed the same inclusion criteria that were used in the previous comparative studies of those guidelines in western countries (Smits et al. 2005; Stiell et al. 2005; Kavalci et al. 2014). Therefore, 142 consecutive patients with mild TBI (GCS 13–15) who were admitted to our institution, the major tertiary care hospital in northeastern Japan, in 2009 and 2010 (6) and who fulfilled the following criteria were included in the current study: (a) recent history (<24 h) of TBI, (b) age ≥17 years, (c) presented at least one of the risk factors stated in CCHR or NOC (Table 1), (d) initial CT performed within 24 h after injury.

**Table 1:** Original version of CCHR and NOC

*1(A) Canadian CT head rule*
Computed tomography is only required for patients with minor head injury with any 1 of the following findings: patients with minor head injury who present with a Glasgow Coma Scale score of 13–15 after witnessed loss of consciousness, amnesia, or confusion
High risk for neurosurgical intervention
1. Glasgow Coma Scale score lower than 15 at 2 h after injury
2. Suspected open or depressed skull fracture
3. Any sign of basal skull fracture
4. Two or more episodes of vomiting
5. 65 years or older
Medium risk for brain injury detection by computed tomographic imaging
6. Amnesia before impact of 30 or more minutes
7. Dangerous mechanism
From Stiell et al. (2011)
*1(B) New Orleans criteria*
Computed tomography is required for patients with minor head injury with any 1 of the following findings. The criteria apply only to patients who also have a Glasgow Coma Scale score of 15
1. Headache
2. Vomiting
3. Older than 60 years
4. Drug or alcohol intoxication
5. Persistent anterograde amnesia (deficits in short-term memory)
6. Visible trauma above the clavicle
7. Seizure
From Haydel et al. (2001)

The demographic data of the 142 patients included age (mean 50 ± 21.7 years; range 17–88 years), sex [96 male (67.6 %) and 46 female (32.4 %) patients], and means of accidents [traffic accident in 68 (47.9 %), falls in 63 (44.4 %), and others in 11 (7.7 %) patients]. On admission to the emergency department, their GCS were 13 in 30 patients (21.1 %), 14 in 45 patients (31.7 %) and 15 in 67 (47.2 %) patients. None of our patients had a penetrating brain injury.

This study was approved by our institutional review board. The requirement for patients’ provision of informed consent was waived.

### Guidelines: the Canadian CT head rule (CCHR) versus the New Orleans Criteria (NOC)

#### The Canadian CT head rule

The following 7 clinical items included in the CCHR (Table 1A) were sought for each patient: GCS < 15 at 2 h after admission, suspected open or depressed skull fracture, any sign of basal skull fracture, vomiting >2 times, age >65 years, retrograde amnesia >30 min, and dangerous mechanism (Stiell et al. 2001).

#### New Orleans Criteria

In all patients, the following 7 NOC clinical items (Table 1B) were sought: headache, vomiting, seizure, intoxication (alcohol, drug), anterograde amnesia, age >60 years, or injury above the clavicles (Haydel et al. 2000). The presence of “intoxication” was defined clinically by evidence of slurred speech, alcoholic fetor, or nystagmus (Smits et al. 2005). We did not include laboratory data because our institution does not perform routine blood toxicology tests in all TBI patients.

### Definition of scoring systems: Canadian score and New Orleans score

**Fig. 1:**
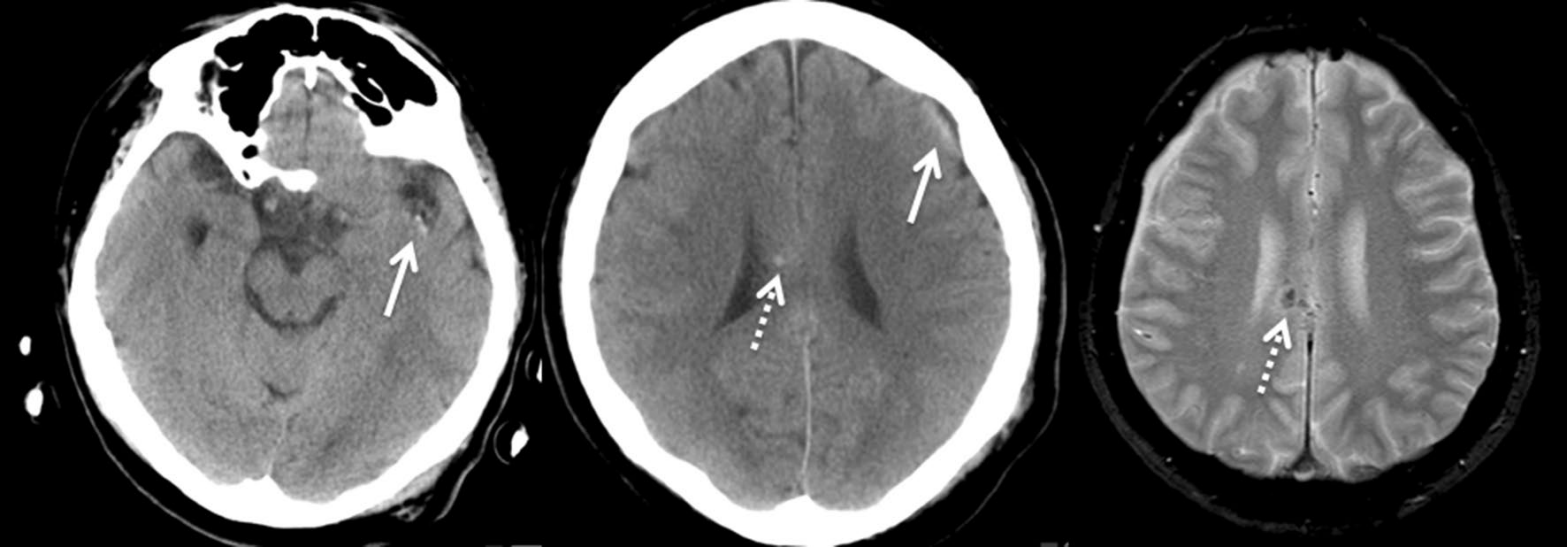
A 42-year-old man who was hit by a car while riding a bicycle. His GCS on admission to the emergency room was 14 out of 15. Two hours after admission, the patient’s GCS remained the same. The patient showed any of the New Orleans guideline’s items, therefore, his New Orleans score was 0. This is in contrast to the Canadian guideline, in which the patient had a GCS score of less than 15 at 2 h after admission (scored +1) and his mechanism of accident (car versus bicycle) fits the dangerous mechanism item (scored +1), leading to a Canadian Score of 2. On CT, the patients shows acute subarachnoid hemorrhages in the left Sylvian fissure (*left panel*, *arrow*) and on the surface of the left frontal lobe (*middle*, *arrow*). More importantly, some hyperdense foci that are suspected to represent a diffuse axonal injury are seen in the corpus callosum (*middle panel*, *dashed arrow*). The follow-up MRI study performed later, confirms the presence of DAI lesions on T2*WI (*right panel*, *dashed arrow*)

As the CCHR and the NOC each contains 7 clinical items (Table 1A, B, respectively), we developed two scoring systems, each composed of 8 grades (0–7): the Canadian score from the CCHR and the New Orleans score from the NOC. In both scoring systems, a patient’s score represented a sum of the number of positive clinical items, each of which was rated +1 if present. Subsequently, Canadian and New Orleans scores were assigned to each patient (Fig. 1).

### CT evaluation

Blinded to clinical data, two neuroradiologists independently reviewed initial screening CT for important CT findings that was defined as any acute brain finding revealed on CT that would require hospital admission or neurosurgical follow-up (Smits et al. 2005; Stiell et al. 2005; Papa et al. 2012). Consensus was used to solve disagreement between readers. Based on the definition by Stiell et al. (2001, 2005), all brain injuries noted on CT were considered clinically important unless the patient was neurologically intact and had 1 of the following lesions on CT: (a) solitary contusion less than 5 mm in diameter, (b) localized subarachnoid bleed less than 1-mm thick, (c) smear subdural hematoma less than 4-mm thick, (d) isolated pneumocephaly, or (e) closed depressed skull fracture not through the inner table.

Based on CT findings, patients were divided in two groups: those with and without important CT findings.

### Analysis and statistics

First, we calculated the sensitivity and the specificity of the CCHR and the NOC for predicting important CT findings in all mild TBI (GCS score 13–15) group (n = 142) and (b) in GCS-15 group (n = 67) to test the reliability of our relatively small and single institution data. When we confirmed that the results of the sensitivity and specificity yielded by the CCHR and the NOC using our data were consistent with those reported in much larger, prospective, multicentric western populations (Smits et al. 2005; Stiell et al. 2005), we proceeded to the analysis of clinical items included in either guideline using the newly introduced scoring systems, as described below.

Originally, the CCHR was introduced for patients with mild TBI with a GCS score of 13 to 15 (Haydel et al. 2000), whereas the NOC were introduced for only those patients with a GCS score of 15, on the premise that all patients who scored 13 or 14 should undergo screening CT. Recently, the NOC were applied to patients with GCS of 13–15 (Smits et al. 2005). In order to treat the Canadian score and the New Orleans score equally during their assessment, we compared them as follows: (a) in all mild TBI (GCS score 13–15) group (n = 142) and (b) in GCS-15 group (n = 67) that constitute the clinical scenario in which the CCHR and the NOC were devised, respectively.

In both clinical settings [(a) and (b)], first, we examined whether the Canadian and New Orleans scores were related to important CT findings by univariate analysis using Mann–Whitney U test. Second, to compare the performance of the two scoring systems in predicting important CT findings, we applied two tests: multiple logistic regression with important CT findings “present” or “absent” serving as the dependent variable, and the two scoring systems as independent variables, and we generated the areas under the receiver operating characteristic curve (AUC) to quantify the comparative performance of the two scoring systems in predicting important CT findings.

In order to determine which of the 14 clinical items (7 items in each guideline) independently predicts important CT findings, we used univariate (Fisher exact test) and multiple logistic regressions. Of all 142 patients with GCS scores of 13–15, 49 (34.5 %) patients showed important CT findings. To maintain statistical power for multiple logistic regressions, the minimum number of events per independent variable should be set as 10 (Novikov et al. 2010; Peduzzi et al. 1996). In that sense, we applied a technique that included in the multiple logistic regressions only those of the 14 clinical items that showed a *P* value ≤0.20 in the univariate analysis.

Of the 67 patients included in the GCS-15 group, 14 (20.8 %) patients showed significant CT findings, which is under the minimum number of dependent events needed to apply multiple logistic regression (Novikov et al. 2010; Peduzzi et al. 1996). Therefore, the independent items predicting important CT findings were not sought in this group. The statistical analyses were performed using the JMP Pro software (ver. 10; SAS Institute, Inc., Cary, NC, USA) and P values <0.05 indicate statistical significance.

## Results

### Clinically important CT findings

Of 142 mild TBI patients, 49 (34.5 %) showed important CT findings. Patients with intraventricular hemorrhage/subarachnoid hemorrhage [32 patients (65.3 %)] and brain contusion [22 (44.9 %)] showed the first and second highest prevalence, respectively. In decreasing order, the next more common important CT findings were: skull fracture [16 patients (32.7 %)], subdural hematoma [15 (30.6 %)], epidural hematoma [3 (6.1 %)], midline shift [3(6.1 %)], and basal cistern compression [2 (4.0 %)]. Twenty-four out of 49 patients (49 %) had more than one important CT finding.

### Relationship demographic data and important CT findings

Patients’ characteristics with respect to important CT findings are shown is Table 2.

**Table 2:** Baseline characteristics of patients

Parameters	Important CT findings present (n = 49)	Important CT findings absent (n = 93)	P value
Age^a^	60.8 (± 20.6)	44.3 (± 20.2)	<.0001*
Sex			0.0022*
Male	25 (51)	71(76.3)	
Female	24 (49)	22 (23.4)	
Mean of accident			0.0030*
Traffic accident	20 (40.8)	48 (51.6)	
Fall	28 (51.1)	31(33.3)	
Others	1 (20.4)	14 (15.1)	
Neurosurgery	4 (8.2)	0 (0)	0.0052*

Unless otherwise indicated, data are numbers of patients, and numbers in parentheses are percentages

* Statistically significant value

^a^Mean (SD) in year (s)

Patients who showed important CT findings had significantly higher age (P = <0.0001), female sex (P = 0.0022) and fall as mechanism of injury (P = 0.0030).

Of the 142 patients, neurosurgical intervention (craniotomy for hematoma evacuation) was performed in 4 (2.8 %). None of the 4 operated patients had GCS = 15 (GCS = 14 in 3 patients, and GCS = 13 in one patient). The indication of surgery was hemorrhagic contusion (in 3 patients) and EDH (in one patient = 1).

### Sensitivity and specificity of CCHR and NOC

In all patients with mild TBI (GCS score 13–15, n = 142), the CCHR showed lower sensitivity (89.8 %), higher specificity (24.7 %) and higher accuracy (47.2 %) in identifying important CT findings compared with the NOC (sensitivity 97.9 %; specificity 9.7 %; accuracy = 40.1 %) (Additional file 1: Table S1).

When limited to the GCS-15 group, both the CCHR and the NOC had equal sensitivity (92.9 %) in identifying important CT findings, but the CCHR showed a slightly higher specificity (22.6 %) and higher accuracy (37.3 %) than the NOC (specificity = 17 %; accuracy = 32.8 %) (Additional file 2: Table S2).

### Comparison of the two scoring systems in GCS-13 to 15 group (n = 142)

Both the Canadian and New Orleans scores showed significant relationships to important CT findings (*P* < 0.0001 and *P* = 0.0063, respectively) in univariate analysis. However, in multivariate analyses, only the Canadian score (*P* = 0.0130) was a predictor of important CT findings (New Orleans score, *P* = 0.6584). Furthermore, the AUC was higher in the Canadian score (AUC = 0.69) than in the New Orleans score (AUC = 0.63).

### Comparison of the two scoring systems in GCS-15 group (n = 67)

When limited to the GCS-15 group for which the NOC were originally designated, only Canadian score showed a positive association with important CT findings in univariate (Canadian: *P* = 0.0043; New Orleans: *P* = 0.09) and multivariate analyses (Canadian, *P* = 0.0128; New Orleans, *P* = 0.69). Furthermore, the AUC was higher in the Canadian score (AUC = 0.73) than in the New Orleans score (AUC = 0.63).

### Clinical items associated with important CT findings

The results of the univariate logistic regressions of the 14 clinical items included in the two guidelines are shown in Table 3. The result of multiple logistic regressions is shown in Table 4A, B.

**Table 3:** Relationship between the 14 clinical items and clinically important CT finding by Univariate analysis

Clinical items	Important finding positive patients [n = 49 (34.5 %)]	Important finding negative patients [n = 93 (65.5 %)]	Fisher exact test P value
(A) NOC			
Headaches (n = 64)	25	39	0.3754
Vomiting (n = 9)	1	8	0.1638^¶^
Seizure (0)	0	0	1.000
Intoxication (n = 31)	11	20	1.000
Anterograde amnesia (n = 20)	7	13	1.000
Aged >60 (n = 52)	30	22	<0.0001*^,¶^
Visible trauma above the clavicle (n = 105)	40	65	0.1608^¶^
(B) CCHR			
GCS < 15 at 2 h post injury (n = 25)	15	10	0.0050*^,¶^
Suspect open or depressed skull fracture (n = 46)	20	26	0.1343^¶^
Any sign of basal skull fracture (n = 19)	10	9	0.1174^¶^
Vomiting >2 times (n = 4)	1	3	1.000
Aged ≥65 (n = 43)	26	17	0.0001*^,¶^
Retrograde amnesia (n = 15)	6	9	0.7748
Dangerous mechanism (n = 73)	26	47	0.8603

* Statistically significant

^¶^Variable that showed a P value of equal or less than 0.20 and which were included in the multiple logistic regression

**Table 4:** Clinical items independently predicting important CT finding in multiple logistic regressions

Clinical items	OR	95 % CI (lower–upper quintile)	P value
(A) With age >60			
Vomiting	3.7	0–1.8	0.20
Visible trauma above the clavicle	1.6	0.6–4.9	0.37
GCS < 15 at 2 h post injury	3.8	1.4–10.8	0.0098*
Suspect open or depressed skull fracture	1.7	0.7–4.4	0.22
Any sign of basal skull fracture	1	0.3–3.6	0.93
Aged >60	6	2.7–14.9	<0.0001*
(B) With age 65 or older			
Vomiting	4.2	0.6–85	0.15
Visible trauma above the clavicle	1.5	0.5–4.4	0.46
GCS < 15 at 2 h post injury	3.8	1.4–10.9	0.008*
Suspect open or depressed skull fracture	1.6	0.6–3.9	0.31
Any sign of basal skull fracture	1	0.3–3.6	0.84
Aged 65 or older	6	2.6–14.6	<0.0001*

* Statistically significant

Based on our preset criterion to input only items which showed a P value of <0.20 in the multiple logistic regression models, the following items were eligible: vomiting, age >60, injury above the clavicles, GCS score <15 at 2 h, suspected open or depressed skull fracture, any sign of basal skull fracture, and age >65. However, as items “age >60” from the NOC and “age >65” from the CCHR substantially overlap, we chose to input only one of the two items at a time, resulting in six variables used in the multiple regression model. Next, we reran the model using the alternative age item. Of the six items input in the multivariate analyses, the age item (“age >60” or “age >65”) (OR 6; *P* < 0.0001, for both) was the strongest independent predictor of important CT findings in either run. The “GCS score <15 after 2 h” item, which is included in the CCHR, was the next independent predictor of important CT findings (OR 3.8, *P* = 0.008 when adjusted for age ≥60; OR 3.8, *P* = 0.0098 when adjusted for age ≥65).

## Discussion

In this single-institution study in Japan, we found that in patients with GCS 13–15 group, the CCHR had a relatively lower sensitivity but a higher specificity than the NOC for important CT findings. In patients with GCS-15 group, the CCHR and the NOC had equally high sensitivities, but the CCHR had a relatively higher specificity for important CT findings. These results were consistent with the previous large-scale western studies, which stipulated that the higher specificity of the CCHR could reduce unnecessary CT scans (Smits et al. 2005; Stiell et al. 2005).

Moreover, by introducing the two scoring systems derived from the two guidelines, we sought to compare them by weighing the contribution of individual clinical items to the overall performance of each guideline. In the GCS 13–15 group, we found that both Canadian and New Orleans scores were significantly associated with important CT findings by univariate analysis, which is consistent with the existing literature showing the usefulness of those guidelines (Smits et al. 2007). More importantly, the superiority of the Canadian score over the New Orleans score in our study by multivariate and ROC analyses agreed with the clinical recommendation of using the CCHR rather than the NOC as reported in the previous western large scale studies (Smits et al. 2005; Stiell et al. 2005). Even in the GCS-15 group, which represents the clinical scenario in which the NOC were devised, the New Orleans score was not associated with important CT findings in univariate and multivariate logistic regression, whereas the Canadian score showed statistically significant values in both tests. In addition, the AUC of the Canadian score was higher than that of the New Orleans score. Our results also confirmed the appropriateness of using the CCHR rather than the NOC even when limited to patients with GCS scores of 15, as shown in the literature (Smits et al. 2005; Stiell et al. 2005). To our knowledge, this is the first Asian study that showed the superiority of the CCHR over the NOC.

How could the CCHR be superior to the NOC? Our analysis of the 14 clinical items that predict important CT findings could provide an explanation. We found that the age item (“age >60” or “age >65”), included in both guidelines, was the strongest independent predictor of important CT findings, consistent with the previous literature (Stiell et al. 2001; Haydel et al. 2000; Smits et al. 2007). However, the remaining independent predictor of important CT findings, the item “GCS score <15 after 2 h,” is only included in CCHR. We presume that the clinical significance of the item “GCS score <15 after 2 h” was related to the fact that it was the only item assessed twice: on admission and 2 h later. In that sense, it could accurately represent the neurological change within a short period during the acute phase of TBI (Fig. 1). Indeed, the items “age >60” and “GCS score <15 after 2 h” represent not only a risk for having lesions on CT, but interestingly, a risk for requiring neurosurgery as well in the original version of the CCHR (Items #1–5, Table 1A). Although, when present, the items “age >60” and “GCS score <15 after 2 h” were rated +1 as others in this study because the presence of each of any item contained in both guidelines indicates CT study for the patient (Smits et al. 2005), our results and those of previous reports reveal their severe clinical significance after a mild TBI (Stiell et al. 2001).

A key question remains why these two guidelines should be additionally assessed in Japan? A previous paper indicated that number of CT scanners per million population in Japan is 3.7 times that for all healthcare level 1 countries and annual X-ray frequency (per 1000 population) in Japan is much higher than that found in USA, Netherlands or Canada where large scale studies on these guidelines have been done (González and Darby 2004). From these statistics, we presume that unlike in Japan where presumably most of patients with mild TBI receive head CT; in western countries, some group of mild TBI patients who actually fulfilled either criterion did not undergo CT, which may lead to some unnoticed selection bias. Thus we believe that two guidelines should also be validated in Japan.

As compared to other western multi-institutional studies, the prevalence of important CT findings was higher in our series. This might be explained by the patients background; actually in our series included patients were older (mean age = 50 years) than in other series (Smits et al. 2005; Stiell et al. 2005) (38.4 and 41 years, respectively). In addition, the age of patients with important CT findings was significantly older than that of patients without important CT findings. This is consistent with the literature, including in our present study, which reports higher age as risk factor for important CT findings (Stiell et al. 2001; Haydel et al. 2000; Smits et al. 2007). Our results add to existing literature that not only older age, but also the female sex and fall as mechanism of accident are significant risk factor for presence of important CT finding on screening CT in patients with mild TBI.

Our study had some limitations. First, our data came from a single hospital. Therefore, some of our results could be influenced by our TBI management protocol. Second, the retrospective design could affect the accuracy of some clinical items collected in this study. However, at our institution, the medical record in the emergency room has a template that contains anatomical diagrams wherein emergency doctors have to mark the location of any traumatic lesions. Therefore, using items’ definition as proposed by their authors (Stiell et al. 2001; Haydel et al. 2000), the following clinical items were easily extracted from the patients’ medical records: “suspected open or depressed skull fracture,” “any sign of basal skull fracture,” and “injury above the clavicles.” Additionally, at our institution the following risk factors are systematically assessed in all TBI patients: mechanism of injury, drug or alcohol intoxication, vomiting, and headaches. The item that was relatively difficult to extract was “amnesia.” We chose to disregard this variable when it was not possible to distinguish whether it was retrograde or anterograde amnesia because they are exclusively included in either guideline. In that sense, we believe that we have as much as possible reduced the bias due to the retrograde design. Third, we have a relatively small number of patients, which could affect the statistical power of the results of our multiple logistic regressions. Therefore, we think that these findings should be confirmed by a large-scale study using a similar methodology.

## Conclusion

In a single tertiary referral hospital in Japan, we introduced two new scoring systems developed from the NOC and the CCHR, and found that the overall performance of the CCHR was superior to that of the NOC in patients with mild TBI. Our results also indicate that CCHR can result in reducing unnecessary CT scans, specifically when limited to patients with a GCS score of 15. These findings are consistent with most of previous large scale western studies that used different approaches.

## Additional files

10.1186/s40064-016-1781-9. Performances of New Orleans Criteria and Canadian Rule in predicting Important CT Findings in all mild TBI patients (n = 142).
10.1186/s40064-016-1781-9. Performances of New Orleans Criteria and Canadian Rule in predicting Important CT Findings when limited to patient with GCS of 15 (n = 67).
