# Supplementary material for: Canadian CT head rule and New Orleans Criteria in mild traumatic brain injury: comparison at a tertiary referral hospital in Japan
Source: Springerplus. 2016 Feb 25;5:176. doi: 10.1186/s40064-016-1781-9 (PMC4766169; doi:10.1186/s40064-016-1781-9)
Supplement: Supplementary file 1 — 10.1186/s40064-016-1781-9. Performances of New Orleans Criteria and Canadian Rule in predicting Important CT Findings in all mild TBI patients (n = 142). [file 40064_2016_1781_MOESM1_ESM.docx]

**Table S1: Performances of New Orleans Criteria and Canadian Rule in predicting Important CT Findings in all mild TBI patients (n=142)**

*A: Canadian CT head Rule*

|  | | Important CT findings | | Total |
| --- | --- | --- | --- | --- |
|  |  | Positive  (n=49) | Negative  (n=93) |  |
| CCHR | Positive:  (n=113) | 44 | 70 | 114 |
|  | Negative:  (n=29) | 5 | 23 | 28 |
| Total | | 49 | 93 | 142 |

*CCHR= Canadian CT head rule.*

- Sensitivity=44/49=89.8%
- Specificity=23/93=24.7%
- Accuracy=23+44/142=47.2%

*B: New Orleans criteria*

|  | | Important CT findings | | Total |
| --- | --- | --- | --- | --- |
|  |  | Positive  (n=49) | Negative  (n=93) |  |
| NOC | Positive:  (n=132) | 48 | 84 | 132 |
|  | Negative:  (n=10) | 1 | 9 | 10 |
| Total | | 49 | 93 | 142 |

NOC: New Orleans Criteria.

- Sensitivity=48/49=97.9%
- Specificity=9/93=9.8%
- Accuracy=9+48/142=40.1%
