# Supplementary material for: Canadian CT head rule and New Orleans Criteria in mild traumatic brain injury: comparison at a tertiary referral hospital in Japan
Source: Springerplus. 2016 Feb 25;5:176. doi: 10.1186/s40064-016-1781-9 (PMC4766169; doi:10.1186/s40064-016-1781-9)
Supplement: Supplementary file 2 — 10.1186/s40064-016-1781-9. Performances of New Orleans Criteria and Canadian Rule in predicting Important CT Findings when limited to patient with GCS of 15 (n = 67). [file 40064_2016_1781_MOESM2_ESM.docx]

**Table S2: Performances of New Orleans Criteria and Canadian Rule in predicting Important CT Findings when limited to patient with GCS of 15 (n=67)**

| CCHR | Important CT Findings | | Total |
| --- | --- | --- | --- |
|  | Positive | Negative |  |
| Positive | 13 | 41 | 44 |
| Negative | 1 | 12 | 13 |
| Total | 14 | 53 | 67 |

**A: Canadian CT head rule**

- **Sensitivity=13/14=92.8%**
- **Specificity=12/53=22.6%**
- **Accuracy=25/67=37.3%**

**B: New Orleans Criteria.**

| NOC | Important CT Findings | | Total |
| --- | --- | --- | --- |
|  | Positive | Negative |  |
| Positive | 13 | 44 | 57 |
| Negative | 1 | 9 | 10 |
| Total | 14 | 53 | 67 |

- **Sensitivity=13/14=92.8%**
- **Specificity=9/53=17.0%**
- **Accuracy=13+9/67=32.8%**
